# Pitfals in recognition and management of trigeminal neuralgia

**DOI:** 10.1186/s10194-020-01149-8

**Published:** 2020-06-30

**Authors:** F. Antonaci, S. Arceri, M. Rakusa, D. D. Mitsikostas, I. Milanov, V. Todorov, M. Cotta Ramusino, A. Costa, On behalf of Headache and Pain Scientific Panels of the European Academy of Neurology (EAN)

**Affiliations:** 1 IRCCS Mondino Foundation, Pavia, Italy; 2 Department of Brain and Behavior, University of Pavia, via Mondino 2, 27100 Pavia, Italy; 3 Department of Neurology, University Medical Centre, Maribor, Slovenia; 4 First Neurology Department, National and Kapodistrian University of Athens, Athens, Greece; 5 St. Naum Hospital of Neurology and Psychiatry, Medical University, Sofia, Bulgaria

**Keywords:** Trigeminal neuralgia, Misdiagnosis, Therapeutic errors, Guidelines

## Abstract

**Background:**

Trigeminal neuralgia (TN) is a severe, disabling form of painful cranial neuropathy. Even though TN has a typical clinical picture, diagnosis it is often missed or delayed in clinical practice. In order to investigate the occurrence of diagnostic and therapeutic errors in TN, we studied 102 patients suffering from TN recruited through a multicentric survey.

**Methods:**

We performed a Pubmed database search on errors and pittfalls in TN diagnosis and management. Then, patients with TN were consecutively enrolled in the period from February 2017 to October 2019, by several European Headache Centers participating in the study, following a call of the Headache and Pain Scientific Panels of the European Academy of Neurology (EAN). Diagnosis of Classical Trigeminal Neuralgia (CTN) was made according to the International Headache Society (IHS) criteria (Tölle et al., Pain Pract 6:153-160, 2006). All the patients were evaluated using telephone/frontal interviews conducted by headache/pain specialists using an ad hoc questionnaire.

**Results:**

A number of 102 patients were recruited, mostly females (F:M ratio 2.64:1). Eighty-six percent of the patients consulted a physician at the time they experienced the first pain attacks. Specialists consulted before TN diagnosis were: primary care physicians (PCP) (43.1%), dentists (in 30.4%), otorhinolaryngologists (3.9%), neurosurgeons (3.9%), neurologists or headache specialists (14.7%), others (8%). The final diagnosis was made mainly by a neurologist or headache specialist (85.3%), and the mean interval between the disease onset and the diagnosis made by a specialist was 10.8 ± 21.2 months. The “diagnostic delay” was 7.2 ± 12.5 months, and misdiagnoses at first consultation were found in 42.1% of cases. Instrumental and laboratory investigations were carried out in 93.1% of the patients before the final diagnosis of TN.

**Conclusion:**

While TN has typical features and it is well defined by the available international diagnostic criteria, it is still frequently misdiagnosed and mistreated. There is a need to improve the neurological knowledge in order to promptly recognize the clinical picture of TN and properly adhere to the specific guidelines. This may result in a favorable outcome for patients, whose quality of life is usually severely impaired.

## Introduction

Trigeminal neuralgia (TN) is a severe, disabling form of painful cranial neuropathy. According to the beta version of the 3rd edition of the International Classification of Headache Disorders (ICHD-3 Beta), TN is “characterized by recurrent unilateral brief electric shock-like pains, abrupt in onset and termination, limited to the distribution of one or more divisions of the trigeminal nerve and triggered by innocuous stimuli. It may develop without apparent cause or be a result of another diagnosed disorder” [1]. TN is classified as idiopathic when it recognizes no apparent cause, classical when it is caused by vascular compression of the trigeminal nerve root, and secondary mainly when it is caused by demyelinating lesions (e.g. multiple sclerosis) or space occupying lesions [2]. The diagnosis of TN requires the absence of a clinically evident neurological deficit, such as hypoaestesia or hypoalgesia occurring in trigeminal regions, that may be indicative of a trigeminal neuropathy. The clinical manifestations of TN usually involve the second and third branch of the trigeminal nerve and the pain is unilateral, although rare cases with bilateral involvement have been reported [3]. A typical aspect of paroxysmal attacks is the refractory period in which the pain cannot be evoked. The intensity of the attacks produces a psychosocial dysfunction significantly impairing quality of life [4, 5]. and for this reason these patients require a prompt diagnosis followed by an appropriate treatment [6, 7]. Furthermore, the suboptimal neuropathic pain management contributes to the significant association between pain severity and poorer health status [7]. However, even though TN has a typical clinical picture, diagnosis is often missed or delayed in clinical practice. There are several reports in literature on diagnostic and therapeutic errors [8, 9], as well as on mismanagement [7] and medication misuse [5] in this condition With this in mind, we designed a multicenter hospital-based study in order to investigate the approach towards TN sufferers, and the diagnostic and therapeutic errors along the temporal pattern of the disease.

## Methods

As in a previous study by our group [10] we performed a Pubmed database search using the following combination of terms: trigeminal neuralgia AND errors OR pitfalls OR misconception OR delay OR mismanagement OR misdiagnosis OR underdiagnosed. We found several peer-reviewed scientific contributions to the field [8, 9, 11, 12].

Patients were then enrolled consecutively in the several Headache Centers involved in the study. Diagnosis of CTN was made according to the International Headache Society (IHS) criteria [1]. In the period from February 2017 to October 2019, 102 patients were recruited on first consultation or follow-up by experts participating in the study (most of patients in Italy, then Bulgaria, Greece, Slovenia, Egypt, Serbia, Albania, Denmark).

The study was officially discussed and approved in Amsterdam by the Pain Panel of the European Academy of Neurology (https://www.eanpages.org/2017/09/16/activities-of-the-ean-scientific-panel-pain-20162017/), in the text there is a link to a pdf version of the questionnaire and to an on line survey The link was published also in the site of the SP Pain and SP Headache at that time and in the web page of the Italian Society for the Study of Headache. This dedicated call was open to neurologist or expert in the field of Headache and facial neuralgia who could have access to the information.

**Fig. 1:**
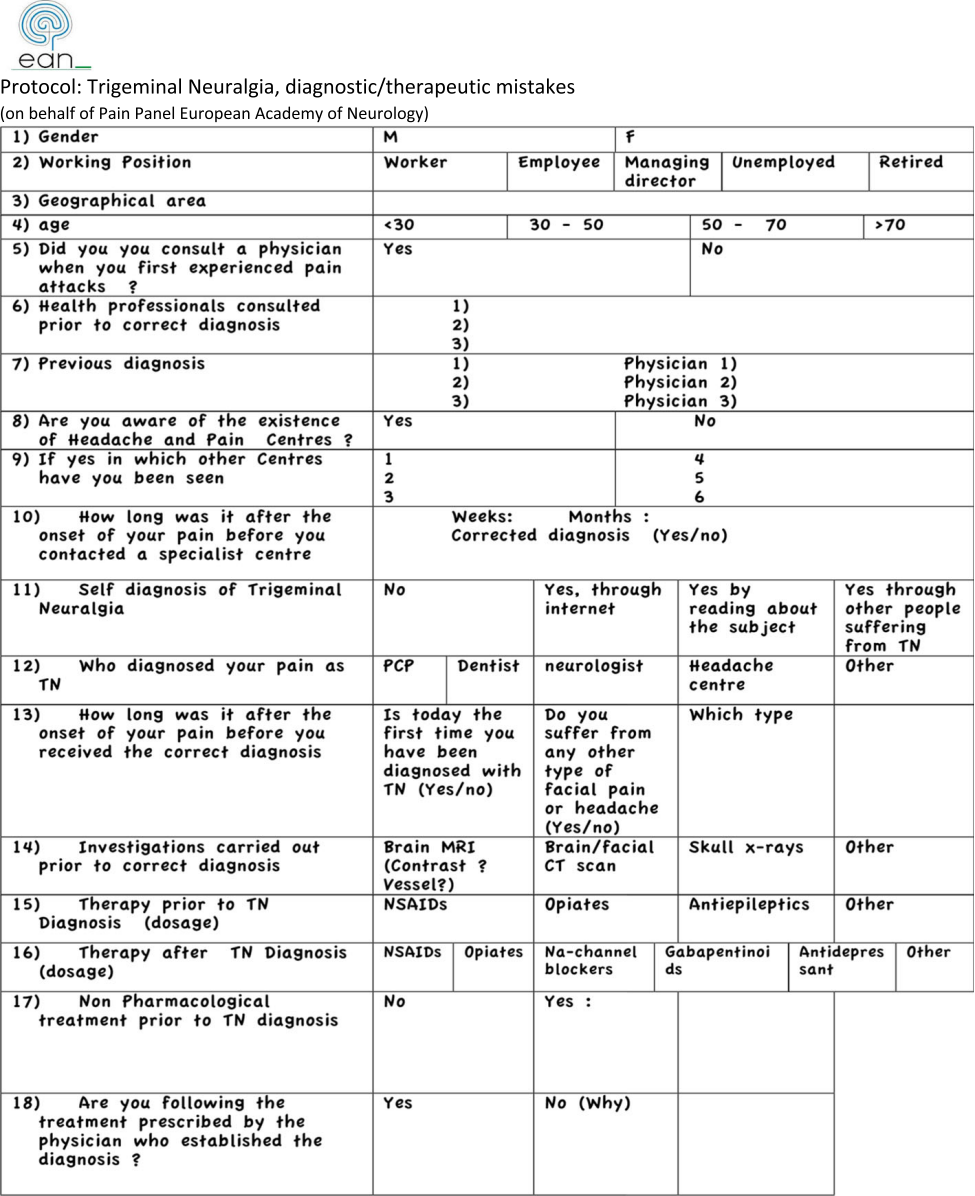
Trigeminal Neuralgia Questionnaire. Protocol: Trigeminal Neuralgia, diagnostic/therapeutic mistakes (on behalf of Pain Panel European Academy of Neurology)

The patients were invited to take part in a face to face or telephone interview conducted by a qualified headache specialist, using an ad hoc questionnaire (Fig. 1) that was the result of a dedicated consensus meeting of the members of the Pain Panel of the European Academy of Neurology (EAN). It was a 18-item questionnaire designed to assess the demographic data, the type of specialist consulted, the time elapsed between the first attacks and the first diagnosis/correct diagnosis, the knowledge (if any) of the existence of dedicated headache centers, the investigations carried out, and any medications prescribed and/or taken. After the questionnaire was administered, the patients interviewed became aware of their CTN diagnosis.

### Statistics

The results were assembled in a database and analyses were carried out using SPSS (version 21.0; SPSS, Chicago, IL, USA).

## Results

### Demographic data

The study included 102 patients with CTN, 74 females and 28 males (F:M ratio 2.64:1). Of these, 1% were less than 30 years old, 23.5% were aged between 31 and 50 years, 50% between 51 and 70 years, and 25.5% were more than 70 years old. One third of patients were retired (33.3%), the rest were employees (49%), unemployed subjects (10.7%), and employers (7%).

### Consulted physicians

**Fig. 2:**
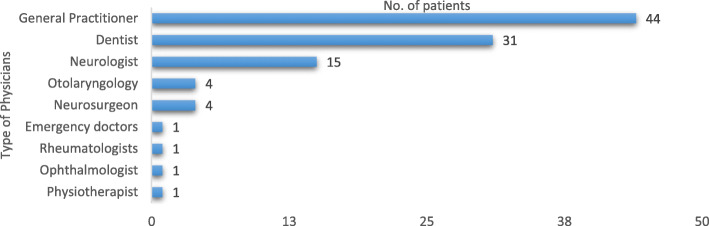
Physicians consulted by the patients before being correctly diagnosed as suffering from TN

Most of patients (*n* = 88, 86.3%) had consulted a physician at the time of the first pain attacks. The remaining patients (*n* = 14, 13.7%) had not sought medical aid after the first episodes (so called “patient delay”). Figure 1 reports the distribution of the physicians consulted at the first visit: approximately half of patients had seen a primary care physician (*n* = 44, 43.1%), and nearly one third a dentist (*n* = 31, 30.4%,). The other consulted specialists were: neurosurgeons (*n* = 4; 3.9%,), otolaryngologists (ENT) (*n* = 4; 3.9%,), ophthalmologists (*n* = 1; 1%,), rheumatologists (*n* = 1; 1%,), emergency doctors (*n* = 1; 1%,), physiotherapist (*n* = 1; 1%). Surprisingly, only 15 patients (14.7%, 2 of which were headache specialists) had referred to a neurologist (Fig. 2). Only 18 patients (17.6%) received a correct diagnosis at the first consultation. It is worth noting that 84 patients (82.4%) consulted a second physician before a correct diagnosis was obtained, 38 patients (37.2%) a third specialist, and 12 patients (11.8%) even a fourth one. The specialists seen on a second consultation were: neurologists (*n* = 41; 48.7%) and headache specialists (*n* = 5; 6,0%), dentists (*n* = 18; 21.4%,) ENT doctors (*n* = 12; 14.2%), primary care physicians (*n* = 3; 3.6%), neurosurgeons (*n* = 2, 2.4%), ophthalmologists (*n* = 2; 2,4%), others (*n* = 1; 1.2%). The third consultation was made by the following: neurologists (*n* = 20; 52.6%,), headache specialists (*n* = 7; 18.4%), ENT doctors (*n* = 5; 13,2%), dentists (*n* = 2; 5.3%), ophthalmologists (*n* = 2; 5.3%), neurosurgeons (*n* = 1; 2.6%) and maxillo-facial surgeons (*n* = 1, 2.6%,). When a fourth consultation had been necessary to reach a diagnosis, the consulted specialist was a neurologist (*n* = 11) and in only one case a general practitioner. One patient, in spite of having received the correct diagnosis of CTN by a neurologist at first consultation, had decided to ask a dentist for a second opinion.

**Fig. 3:**
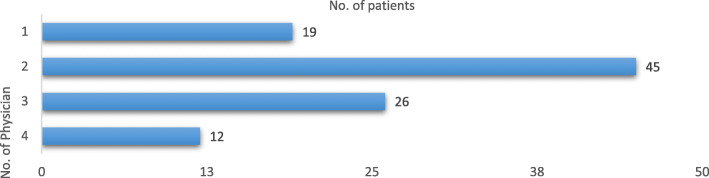
Number of physicians consulted by patients before being correctly diagnosed as suffering from TN

Most of the patients consulted two physicians (*n* = 45; 44.1%) prior to obtain a correct diagnosis, while 26 patients (25.5%) consulted three physicians, 12 patients (11.7%) four physicians, and only 19 patients (18.7%) consulted one physician (Fig. 3). Eighty patients (78.4%) were aware to be likely to suffer from a form of trigeminal neuralgia before submitting the questionnaire. Only few patients (*n* = 22, 21.6%) were diagnosed as suffering with CTN before completing the questionnaire.

We found that generally CTN diagnosis had been made by a neurologist (*n* = 72, 70.6%), or a headache specialist (*n* = 15; 14.7%). In a few cases, diagnosis was received by dentists (*n* = 6; 5.9%), neurosurgeons (*n* = 6; 5.9%), and only in three cases by primary care physicians (*n* = 3; 2.9%). Unfortunately, 49% of the patients interviewed were not aware of the existence of Headache Centers or other specific structures dedicated to the treatment of headache and cranial neuralgias. Furthermore, 18.6% of the interviewed patients claimed to have self-diagnosed CTN on the basis of different sources of information (Internet *n* = 16, scientific books *n* = 1, or talk with other people suffering from CTN, *n* = 2), before seeking medical confirmation. Thirty-one patients (31.4%) in our study also suffered from another type of headache, as diagnosed by a specialist before the onset of their neuralgia (migraine without aura *n* = 18, tension type-headache *n* = 12; cluster headache *n* = 1; Horton’ arteritis *n* = 1).

**Fig. 4:**
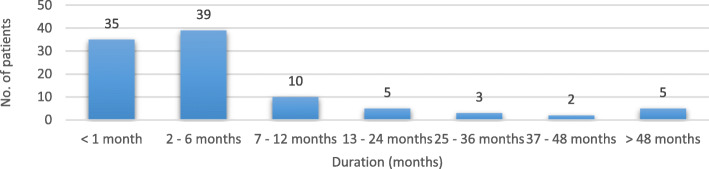
Time elapsed between disease onset and first Headache Center consultation (“Patient Delay”)

**Fig. 5:**
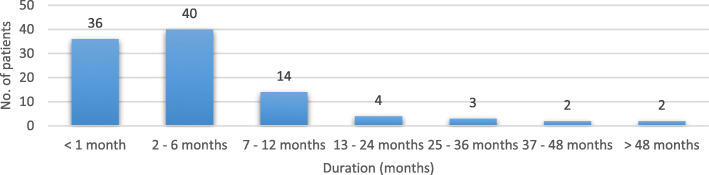
Time elapsed between disease onset and correct diagnosis (“Diagnostic Delay”)

The mean interval between onset of the disease and specialist consultation (“Patient Delay”) by a neurologist or a Headache Center was 10.8 ± 21.2 months (range 0–144 months). In Italy, delay was 8.02 ± 14.2 months, whereas in other European Centers was up to 12,6 ± 25,05 months. In our cohort, 2 outlier patients (Fig. 4) influenced significantly the average delay: one patient was indeed diagnosed after 72 months and another one after as long as 12 years. Three of the interviewed patients did not receive any headache specialist evaluation. The average time between disease onset and a correct diagnosis (“Diagnostic Delay”) was 7.2 ± 12.5 months (in Italy 8.4 ± 12.8 months, in the other European centers 7.13 ± 13.01 months). Only in one patient no information was obtained. Misdiagnoses at first consultation were reported in 43 cases (42.1%), while 40 subjects (39.2%) did not receive a diagnosis during the visit; only 19 subjects (18.4%) received a correct one. Only one patient, despite obtaining the correct diagnosis, asked for a second specialist consultation. In the group of patients (*n* = 84) who underwent a second consultation, 28 patients (33.3%) were misdiagnosed, 11 patient (13.1%) did not receive a definite diagnosis, while 45 patients (53.6%) were not diagnosed correctly, although one received the indication for a nonspecific treatment with corticosteroids. In the group facing a third evaluation (*n* = 38), 6 subjects (15.8%) did not obtain a specific diagnosis, 6 patients received a misdiagnosis, while 26 patients (68.4%) received a correct diagnosis (15.8%) (Fig. 5).

The different diagnoses received before the correct one were also analyzed. The total number of misdiagnoses was 77 (mean number of diagnosis per patient: 0.75). Misdiagnoses on the first consultation were 43, on the second 28, and on the third 6. Overall, the reported misdiagnoses and related frequency were the following: dental problems (*n* = 37, 48%,), including toothache, periodontal abscess, dental caries, dental granulomas; sinusitis (*n* = 11, 14.3%); unspecified facial pain (*n* = 7, 9.1%); unspecified headache (*n* = 6, 7.8%); migraine (*n* = 5, 6.5%); cluster headache (*n* = 4, 5.2%); temporomandibular joint dysfunction (*n* = 3, 3.9%); tension-type headache (*n* = 1, 1.3%), glaucoma (*n* = 1, 1.3%), otitis (*n* = 1 1.3%) tonsillitis (*n* = 1, 1.3%).

### Investigations

Instrumental (neuroimaging or others) and laboratory investigations prior to establish a TN diagnosis had been carried out in almost all cases (95 patients, 93.1%). Most of patients (*n* = 70, 73.7%) had undergone a brain MRI in order to detect a possible neurovascular conflict or other causes of symptomatic TN; 40 patients (42.1%) had undergone a CT scan, 12 patients (12.6%) a skull X-rays, 8 patients (8.4%) an orthopantomography, 8 patients (8.4%) a blink reflex test, 2 patients (2.1%) a spine X-Ray, 2 patients (2.1%) a carotid ultrasound imaging, one patient (1%) an electroencephalogram (EEG). More than one instrumental examination had been performed in 37 patients (38.9%). In 25 patients (26.3%) some investigations (skull X-ray, orthopantomography, EEG, carotid ultrasound imaging, spine X-ray) appeared to be unnecessary [2, 3, 5].

### Treatment

In our sample, 19 patients (18.6%) had not received any symptomatic treatment before diagnosis. The remaining patients had been prescribed symptomatic drugs, especially analgesics, such as NSAIDs (*n* = 69, 67.6%) and opiates (*n* = 19, 18.6%). In the latter group, 13 patients used opiates in association with NSAIDs. Then, 8 patients had been prescribed gabapentinoid drugs (gabapentin 100 mg/day or pregabalin 150 mg/day), 3 patients had been treated with triptans as needed. Other treatments were: antibiotics (*n* = 1), benzodiazepines (*n* = 1), B vitamin supplements (*n* = 2), mannitol i.v. (*n* = 2), verapamil (*n* = 1), duloxetine (*n* = 1), topiramate (*n* = 1). Seven patients claimed to be on treatment with unspecified anti-epileptic drugs.

After the correct diagnosis was established, the first-choice treatment was: carbamazepine in 80.3% (*n* = 82), gabapentinoid drugs in 11.7% (*n* = 12), topiramate in 2% (*n* = 2), lamotrigine in 2% (*n* = 2); oxacarbazepine in 1% (*n* = 1), methylprednisolone in 1% (*n* = 1), opiates in 1% (*n* = 1), antidepressants not better specified in 1% (*n* = 1). In different phases of the disease, some patients switched to another pharmacological regimen, with the addition/substitution of a second drug. In 10 patients (9.8%) the drug introduced was lamotrigine, a choice due to partial/complete inefficacy of the first treatment, but the most frequent treatments chosen as second option were gabapentinoids 24.5% (*n* = 25), in addition/substitution to the first one. The other associated treatments were: unspecified antidepressants 6.8% (*n* = 7); carbamazepine 3.9% (*n* = 4), oxacarbazepine 1.9% (*n* = 2), venlafaxine 1.9% (*n* = 2), duloxetine (*n* = 1). Five unresponsive patients had been treated with amitriptyline as a third choice. It is worth noting that 18 patients received a treatment not included in the dedicated guidelines for TN [13–15], i.e. topiramate (*n* = 2), methylprednisolone iv (*n* = 1), opiates (*n* = 1), amitriptyline (*n* = 5), duloxetine (*n* = 1), unspecified antidepressants (*n* = 8).

Before TN diagnosis, 17.6% of the patients (*n* = 18) had already undergone some treatment procedures, such as a tooth extraction (*n* = 10, 55.5%), in 2 cases even multiple, or treatments not recommended, i.e. acupuncture (*n* = 4, 22.2%); local injection of steroids (*n* = 2, 1.9%), anesthetics, or weakly recommended such as botulinum toxin (*n* = 1), Thecar therapy (*n* = 1). Only 1 patient, seen as a new referral, had undergone 3 subsequent microvascular decompression surgeries, and later a balloon compression and a percutaneous glycerol rhizolysis of Gasserian ganglion. Eighty-two patients (80.4%) had followed the therapy prescribed after the diagnosis, whereas 20 subjects (19.6%) had interrupted the treatment, due to the following reasons: side effects (in particular dizziness and asthenia) (*n* = 1), ineffectiveness (*n* = 17), leukopenia (*n* = 1), allergic reaction (*n* = 1).

## Discussion

In the present study, we evaluated the occurrence of misdiagnosis and errors in the management of patients suffering from TN. This issue has also been investigated in the past [5, 7–9, 11, 16], similarly to what done for other types of pain (i.e primary and secondary headaches), with all the studies suggesting that especially in an early phase of disease diagnostic errors are very common. Many cases of TN are therefore incorrectly treated in primary care [17].

On a first analysis, the demographic data of our sample are consistent with previous studies reporting a greater prevalence of TN in females (F: M ratio 2.64: 1) [18]. The evaluation of the data obtained revealed that about half of the patients had received a wrong diagnosis at the first medical evaluation, while a small number had been correctly classified as suffering from TN. Moreover, the mean interval between the onset of symptomatology and the actual diagnosis was found to exceed 7 months. This finding was apparently similar in all the centers involved in the study, suggesting a similar general application of the diagnostic criteria of TN.

Interestingly, we observed that in order to obtain a correct diagnosis, patients had to contact a mean of two specialists. While the latency between the first access of patients to health resources and the correct diagnosis may appear high, it has to be said that the mean diagnostic delay in patients with a primary headache like cluster headache (CH) was reported to be even higher (4.9 years and over) in previous studies [19, 20]. This could be explained by different factors: TN is probably more known than CH in the medical field, and patients suffering from TN report a sharp-triggered pain with daily pattern of several attacks [18].

During their full blown phase of disease, TN patients in our study had consulted several doctors, but in most cases the diagnosis had been made by a neurologist or a headache specialist. This confirms that, while the general practitioner often represents the first medical figure that patients consult at the first presentation of pain attacks, TN remains a strictly specialistic diagnosis. Moreover, in our study we noted that due to the topographic distribution of the pain (II or III branch of the trigeminal nerve) and the characteristics of the pain itself (sharp, stabbing) the first specialist consulted, in the suspicion of a dental pathology, had been the dentist, a well known occurrence in clinical practice [21]. This had resulted in several cases of misdiagnosed underlying pathology, and hence in unnecessary dentistry procedures, such as dental extractions. In this respect, Garvan et al. showed that 73% of patients with TN, in an attempt to control the pain, received unnecessary dental assessment, and of these, more than 60% underwent dental extraction (for a total of 680 teeth extracted) [9]. These data are in line with those later obtained by other authors reporting in a retrospective study that more than 80% of CTN patients had referred to a dentist for new onset trigeminal symptoms, and that 66% of them had received unnecessary dental treatment, including extractions (mean of 2 teeth), root canal treatments and inplants, before consulting a neurologist [12]. However, the delay in the diagnosis also by other specialists implies a mismanagement of the disease. Thus, our data are in agreement with previous studies reporting primary care physicians and dentists as the main figures consulted by the patients for the first time, and neurosurgeons or neurologists very frequently over time [8]. Among pain conditions seen by dentists, orofacial neuralgia following whiplash-associated traumas has also been reported, and it should also be distinguished from TN [22].

It is also interesting to compare these data to those regarding CH, a primary headache form that is frequently misdiagnosed as TN. Our results appear indeed to be in contradiction with a previous study on diagnostic and therapeutic errors in CH where the first specialist consulted was the neurologist (48,6%), the dentist being seen only in 2.8% of the cases, and the misdiagnosis of dental problems had occurred in 4.2% of cases [19]. The different clinical presentation of CH in bouts compared to TN may explain such discrepancy. In another study focused on this issue [20], as much as 25% of CH patients had been diagnosed with TN, in spite of the presence of cranial autonomic symptoms and the typical temporal pattern ot attacks. Another recent survey on the frequency of headache and pain disorders in neurological outpatients showed that the first specialist consulted had been a neurologist in as low as 8.1% of cases [23].

In the present survey, 4% of TN cases were mistaken as a temporo-mandibular joint disorder (TMD). This aspect has been discussed in a previous investigation [11] reporting a high frequency of this diagnostic error in TN patients, attributed by the authors primarily to an often overlooked aspect of TN, i.e. the fact that pain is triggered by use of the jaws (chewing, talking), and to the observation that often TN occurs in older patients and TMD is more common above 50 years of age.

With regard to medical approach, in our study, more than half of the patients (62.7%) had to consult two doctors before receiving the correct diagnosis. As suggested by EAN guidelines on TN [13, 15], a MRI of the brain and brainstem is recommended to exclude a symptomatic form of disease. In our study, 73.7% of patients had undergone a brain MRI study and some of them also a brain CT scan, along with other unnecessary examinations such as orthopanthography, EEG, spine X-rays, skull X-rays, and carotid ultrasound imaging. This patient delay is an aspect already recognized by previous reports: Maarberg et al. found an even longer delay to diagnosis in their patients [3]. The role of pain remission periods, shown especially in an early phase of disease by the majority of patients, may be relevant to explain this discrepancy [24].

It is worth underlining that, before the diagnosis, patients had been formally prescribed or were taking symptomatic drugs according to a self-medication regimen, with little or no benefit, especially from the NSAID class and opioids for acute attacks. There is indeed evidence for a limited effect of opioids in neuropathic pain in general [25]. Only a minority of patients had been prescribed anti-epileptic drugs, of the class of gabapentinoids, before the diagnosis. In addition, 3 patients had been adviced to assume triptans for the acute attack, without benefit. Then, after the correct diagnosis, in agreement with the available guidelines, carbamazepine and oxcarbamazepine were introduced as a first-line treatment. Other drugs used as a first-line therapy, either in single-dose or in combination, were gabapentinoids, lamotrigine and topiramate. These observations are in agreement with current evidence-based guidelines on TN treatment from the EAN which consider carbamazepine as the first-choice treatment for TN [13, 14]. From the analysis of our data, it would therefore appear that the limiting factor in the workup and management of TN was the diagnostic delay.

According to the mentioned guidelines, the indication for surgery is a condition in which pain is not sufficiently controlled medically or a medical treatment is poorly tolerated [15]. Patients should be informed of such possibility at an early stage. Although surgical treatment for TN is generally effective, the important complications of the different procedures limit its use as a first-line option: for example percutaneous procedures of Gasserian lesions can cause facial sensory loss (painful anesthesia). Gamma-knife appears to be the least invasive and the safest procedure, but pain relief may take long to develop [15]. In our patients, drugs not formally recommended for TN, such as amitriptyline, duloxetine, venlafaxine and in one case parenteral methylprednisolone, had been introduced as add-on therapy. However, once the diagnostic workup has been set up and treatments have been prescribed in accordance with to the international guidelines, therapeutic errors should no longer occur [14, 15].

This study has some limitations. First, in spite of an European-based call, the number of recruited patients was low and with a significant predominance of Italian subjects. This may have influenced the results in terms of poor homogeneity of the patient sample, and also in view of a possible different approach to the patients among the different centers. In addition, in evaluating of data, it should be considered that in some countries it is quite difficult to consult a neurologist before having been seen by a general physician, as well as to consult a specialist of a Headache Center before undergoing a neurological assessment. In this respect, in Italy patients can directly ask for a neurological visit or a consultation with an Headache Center specialist. By contrast, unfortunately we could not obtain information as to the management of such patients in the health systems of the other countries involved in this survey. Further, the value of patient delay may have been affected by the existence of a waiting list of neurologists or headache specialists. For instance, at our Institute there is the possibility for patients with trigeminal neuralgia to be seen quickly, in an emergency setting but it is not necessarily so in other structures. This may also explain why the delay observed in our survey, albeit long, was found to be shorter than that reported by other authors [3, 8]. All these aspects may have therefore represented a bias for our study, suggesting the need for replicating a survey on a wider number of patients, and within a more homogeneous investigation setting.

## Conclusions

Even with the above limitations, the overall findings from this survey appear to be consistent with those previously reported by several authors with regard to errors in recognition and management of TN. Our data suggest that while TN has typical features and it is well defined by the available international criteria, it is still frequently misdiagnosed and mistreated. To avoid this, a tight cooperation on the basis of a continuous medical education between neurologists, general practitioners, dentists and neurosurgeons appears to be mandatory when evaluating a patient with orofacial pain suggesting TN. Larger population studies may provide further evidence in order to identify the correct strategies to reduce delays due to both patients and professionals, and to expand our knowledge on the overall management of this disease.

## Data Availability

The datasets used and/or analysed during the current study are available from the corresponding author on reasonable request.

## Abbreviations

TN: Trigeminal neuralgia
CTN: Classical Trigeminal Neuralgia
ENT: Otolaryngologists
